# Relationship Between Smoking and Angina Across Diverse Demographic and Socioeconomic Subgroups in the United States: A Retrospective Study Using the Behavioral Risk Factor Surveillance System (BRFSS) Database

**DOI:** 10.7759/cureus.95442

**Published:** 2025-10-26

**Authors:** Meenakshi R Yathindra, Maheswari Pulluru, Amol Deokar, Faizaan Farukh Vohra, Mahima Kamble, Bhavana Nelakuditi

**Affiliations:** 1 Internal Medicine, Kasturba Medical College, Mangaluru, IND; 2 Internal Medicine, Government Medical College (GMC), Srikakulam, IND; 3 Internal Medicine, Kings College Hospital NHS Trust, London, GBR; 4 Emergency, Sterling Hospital, Vadodara, IND; 5 Internal Medicine, Avalon University School of Medicine, Willemstad, CUW; 6 Internal Medicine, NRI Medical College, Guntur, IND

**Keywords:** angina, brfss, chd, odds ratio, retrospective study, smoking

## Abstract

Introduction: Coronary heart disease (CHD) is a leading cause of morbidity and mortality worldwide and commonly presents as angina. Although smoking has been studied extensively as a modifiable risk factor of CHD, the influence of specific demographic and socioeconomic factors on their relation is less well understood.

Objective: To evaluate the association between smoking and angina or CHD, with particular attention to demographic and socioeconomic factors.

Methodology: A retrospective study was conducted using data from the 2022 Behavioral Risk Factor Surveillance System (BRFSS). Only data with detailed responses to questions about smoking and angina were included. The exposure variable was current smoking status, and the outcome variable was a history of angina or CHD. Demographic variables included gender, ethnicity, and age, whereas annual income and education were included as socioeconomic variables. Statistical analyses were performed to evaluate the relationship between smoking and angina/CHD.

Results: A total of 405,798 respondents’ data were included from 445,132 participants. A significant association was found between smoking and angina, with an odds ratio (OR) of 1.149 and a 95% confidence interval (CI) of 1.106-1.193 when comparing smokers and non-smokers. Higher odds of association were found among young adults (OR = 4.504; CI 2.6523-7.3709), females (OR = 1.356; CI 1.2806-1.435), Hispanic individuals (OR = 1.502, 95% CI 1.3505-1.6668), Black individuals (OR = 1.214, 95% CI 1.0519-1.3967), and individuals with advanced education (OR = 1.186, 95% CI 1.1255-1.2494).

Conclusions: The findings of this study suggest that angina/CHD was reported significantly more frequently among smokers than non-smokers. The stronger associations observed in young adults, females, and Hispanic and Black individuals highlight the need for population-specific targeted interventions.

## Introduction

Angina, typically presenting as chest discomfort, is the primary clinical manifestation of ischemic heart disease, one of the leading causes of global morbidity and mortality. Since chest pain may arise from both cardiac and non-cardiac origins, a thorough clinical assessment, particularly a detailed patient history, is essential [1]. Coronary heart disease (CHD) affects more than 17 million adults in the United States and remains a leading contributor to mortality. Among those diagnosed, approximately 55% are male. CHD is responsible for over 500,000 deaths annually nationwide [2].

Estimates from earlier population-based surveys and healthcare records suggest that angina affects approximately 2% to 7% of middle-aged individuals in high-income countries [3]. Tobacco use has been consistently linked to elevated risks of both morbidity and mortality across various populations. Global projections indicate that annual deaths attributable to smoking are expected to increase from approximately 3 million in 1995 to 10 million by 2030, with an estimated 70% of these fatalities occurring in low- and middle-income countries [4].

Smoking is a major modifiable risk factor for CHD and is commonly reported among patients presenting with angina. Smoking cessation in individuals with a history of acute coronary syndromes, including angina, has been shown to reduce the risk of recurrent myocardial infarction and mortality by 30% to 50% [5].

While several studies have explored the general relationship between smoking and CHD, limited attention has been given to how this association differs across diverse demographic and socioeconomic subgroups within the United States. By analyzing recent nationally representative data from the 2022 Behavioral Risk Factor Surveillance System (BRFSS), this study adds updated evidence on population-specific variations in this relationship, helping identify high-risk groups for targeted preventive strategies.

The aim of this study is to evaluate the association between smoking and angina or CHD by assessing the odds of developing the disease in smokers versus non-smokers, based on the demographic (age, gender, and race) and socioeconomic characteristics (annual income and education levels) of participants.

## Materials and methods

This cross-sectional analytical study used data from the 2022 BRFSS. The Centers for Disease Control and Prevention (CDC) conducts the BRFSS, a nationally representative telephone-based survey, every year in all 50 states and territories of the United States. It gathers self-reported information on health status, behaviors, and healthcare service utilization from adults who are not institutionalized and who are at least 18 years old. The 2022 BRFSS dataset, comprising a total of 405,798 respondents, was used for this analysis.

The study population comprised 405,798 adults who participated in the survey in 2022. Complete responses regarding smoking status and history of angina or CHD were required for inclusion. Participants with missing data for these variables (*n* = 39,334) were excluded from the analysis.

The presence of either CHD or angina, as indicated by participants' affirmative answers when asked if they had ever received a diagnosis from a medical professional, was the outcome variable. Current smoking status was the primary exposure variable, and it was classified as *Yes* for people who reported smoking daily or occasionally and *No* for people who did not currently smoke.

Demographic variables included gender (male or female), race/ethnicity (White non-Hispanic, Black non-Hispanic, and Hispanic/other), and age (categorized as 18-24, 25-44, 45-64, and ≥65 years). Socioeconomic factors included annual income, categorized into two groups: below $50,000 and $50,000 or above, and education level, categorized as basic education (up to high school) and advanced education (some college or more).

Potential confounding and effect modification by age, gender, race/ethnicity, income, and education were explored through stratified subgroup analyses, rather than multivariable adjustment, to highlight population-specific variations in the association between smoking and angina/CHD.

Statistical analysis

Statistical analyses were performed using R software, version 4.3.1 (2023). The prevalence of angina or CHD in smokers and non-smokers was compiled using descriptive statistics. Binary logistic regression was employed to estimate unadjusted odds ratios (ORs) and 95% confidence intervals (CIs) for the association between smoking and angina/CHD, as the outcome variable was dichotomous (presence or absence of disease). Logistic regression is appropriate for modeling binary health outcomes and allows for the estimation of odds while accounting for categorical predictors.

The different demographic and socioeconomic groups were subjected to stratified analyses, and a *P*-value of <0.05 was considered statistically significant. The BRFSS survey weights were applied to account for the complex sampling design, ensuring national representativeness. Weighted analyses also adjusted for differences in response probabilities and demographic distributions.

Ethical consideration

Because the BRFSS dataset is de-identified and publicly available, neither ethical approval nor informed consent was required for this study. All analyses were conducted in accordance with ethical standards for secondary data research.

## Results

In 2022, a total of 445,132 individuals across the United States participated in the BRFSS study. Among them, 405,798 (91.16%) who answered the relevant questions were included in the study, while the remaining 39,334 (8.83%) were excluded.

Table 1 shows the prevalence of angina in smoking and non-smoking groups. Out of the total 49,226 participants who reported smoking, 3,365 (6.8%) had angina. In contrast, among the 356,572 participants who did not report smoking, 21,407 (6%) had angina. The OR of 1.149 indicates that participants who reported smoking were 14% more likely to have angina compared with non-smokers, representing a statistically significant association.

**Table 1:** Prevalence of current smoking among participants with self-reported angina or coronary heart disease. *Significant. CI, confidence interval; OR, odds ratio

Current smoker	Angina or coronary heart disease		OR (CI)
	Yes	No	
Yes	3,365 (6.8%)	45,861 (93.2%)	1.149* (1.1061-1.193)
No	21,407 (6%)	335,165 (94%)	

Table 2 shows the prevalence of angina in smoking and non-smoking groups based on demographic characteristics. The prevalence of angina among participants who reported smoking was 22 (1.4%) in the 18-24 years age group, 211 (1.4%) in the 25-44 years age group, 1,565 (7.4%) in the 45-64 years age group, and 1,567 (13.1%) in participants aged 65 years and above. When gender was considered, angina was more prevalent in males who smoked (1,869, 7.5%) compared to females who smoked (1,496, 6.2%). Among racial groups, White participants who smoked had a prevalence of 2,554 (7.3%), Black participants who smoked had a prevalence of 251 (5.8%), and Hispanic/other-race participants who smoked had a prevalence of 455 (5.3%).

**Table 2:** Prevalence of current smoking among participants with self-reported angina or coronary heart disease, by demographic characteristics. *Significant. CI, confidence interval; OR, odds ratio

Demographic characteristics	Current smoker	N	Participants with Angina or coronary heart disease	Participants without Angina or coronary heart disease	OR (CI)
Age groups					
18-24 years	Yes	1,593	22 (1.4%)	1,571 (98.6%)	4.504* (2.6523-7.3709)
	No	23,234	72 (0.3%)	23,162 (99.7%)	
25-44 years	Yes	14,560	211 (1.4%)	14,349 (98.6%)	2.054* (1.7455-2.4103)
	No	83,438	593 (0.7%)	82,845 (99.3%)	
45-64 years	Yes	21,109	1,565 (7.4%)	19,544 (92.6%)	1.926* (1.8133-2.0437)
	No	114,534	4,573 (4%)	109,961 (96%)	
65+ years	Yes	11,964	1,567 (13.1%)	10,397 (86.9%)	1.111* (1.0503-1.1748)
	No	135,366	16,169 (11.9%)	119,197 (88.1%)	
Gender					
Male	Yes	24,911	1,869 (7.5%)	23,042 (92.5%)	0.987 (0.9383-1.0388)
	No	166,603	12,646 (7.6%)	153,957 (92.4%)	
Female	Yes	24,315	1,496 (6.2%)	22,819 (93.8%)	1.356* (1.2806-1.435)
	No	189,969	8,761 (4.6%)	181,208 (95.4%)	
Race					
White, non-Hispanic	Yes	34,788	2,554 (7.3%)	32,234 (92.7%)	1.111* (1.0635-1.1598)
	No	260,549	17,348 (6.7%)	243,201 (93.3%)	
Black, non-Hispanic	Yes	4,361	251 (5.8%)	4,110 (94.2%)	1.214* (1.0519-1.3967)
	No	26,745	1,281 (4.8%)	25,464 (95.2%)	
Hispanics/Others	Yes	8,542	455 (5.3%)	8,087 (94.7%)	1.502* (1.3505-1.6668)
	No	59,283	2,141 (3.6%)	57,142 (96.4%)	

The likelihood of angina associated with smoking, expressed as an OR, was consistently higher in smokers across all demographic groups. In the 18-24 years age group, the likelihood of angina was 350% higher (OR = 4.504) in smokers, while in the 25-44 years age group it was 105% higher (OR = 2.054). Similarly, in the 45-64 years age group, the likelihood of angina was 92.6% higher (OR = 1.926) in smokers, and in participants aged 65 years and above, it was 11% higher (OR = 1.111). When considering gender, the likelihood of angina was 1.3% lower (OR = 0.987) in male smokers and 35.6% higher (OR = 1.356) in female smokers compared to their non-smoking counterparts.

Among racial groups, the likelihood of angina was 11% higher (OR = 1.11) in White smokers, 21.4% higher (OR = 1.214) in Black smokers, and 50.2% higher (OR = 1.502) in Hispanic/other-race smokers compared to non-smokers. These findings suggest an increased likelihood of angina among smokers across all demographic groups.

Table 3 shows the prevalence of angina in smoking and non-smoking groups based on socio-economic characteristics. Among participants who smoked, the prevalence of angina was 1,648 (7%) in those with basic education and 1,707 (6.7%) in those with advanced education. When income was considered, angina was more prevalent among participants with an annual income of <$50,000 (2,134, 8.4%) compared to those with an income of >$50,000 (745, 4.6%). The likelihood of an association between smoking and angina, expressed as ORs, was 2.4% higher (OR = 1.024) in participants with basic education and 18.6% higher (OR = 1.186) in those with advanced education. Similarly, the likelihood of association was 2.3% higher (OR = 1.023) in participants with an annual income of <$50,000 and 0.9% lower (OR = 0.99) in participants with an annual income of >$50,000.

**Table 3:** Prevalence of current smoking among participants with self-reported angina or coronary heart disease, by socioeconomic characteristics. *Significant. CI, confidence interval; OR, odds ratio

Socioeconomic characteristics	Current smoker	N	Participants with angina or coronary heart disease	Participants without angina or coronary heart disease	OR (CI)
Education level					
Basic education	Yes	23,441	1,648 (7%)	21,793 (93%)	1.024 (0.9678-1.0832)
	No	97,250	6,687 (6.9%)	90,563 (93.1%)	
Advanced education	Yes	25,628	1,707 (6.7%)	23,921 (93.3%)	1.186* (1.1255-1.2494)
	No	257,929	14,637 (5.7%)	243,292 (94.3%)	
Annual income					
Less than $50,000	Yes	25,519	2,134 (8.4%)	23,385 (91.6%)	1.023 (0.9737-1.0752)
	No	111,075	9,094 (8.2%)	101,981 (91.8%)	
Greater than $50,000	Yes	16,291	745 (4.6%)	15,546 (95.4%)	0.991 (0.9166,1.0702)
	No	179,306	8,271 (4.6%)	171,035 (95.4%)	

## Discussion

Smoking and CAD: Risk, mechanisms, and effects

With 445,132 records initially available, our study included 405,498 individuals overall from the BRFSS dataset. Following a predefined criteria exclusion of 39,334 records, the study revealed a statistically significant correlation between current smoking and self-reported angina or CHD. Of those with angina or CHD, 6.8% were current smokers compared to 6% among those free of these diseases. For those with angina or CHD, the OR for being a current smoker was 1.149 (95% CI 1.1061-1.193), meaning a modest but notable higher likelihood of smoking among this subgroup.

In a large multicenter study of patients with acute myocardial infarction (AMI), Buchanan et al. found that smoking status was tightly correlated with angina frequency and health-related quality of life (HRQOL). While consistent smokers suffered worse angina and poorer HRQOL, never smokers had the best outcomes. Former smokers and those who quit following AMI showed better HRQOL; recent quitters had mental health equivalent to never smokers. Through oxidative stress, inflammation, platelet activation, and vasospasm, smoking exacerbates CAD and angina. Importantly, quitting smoking after an AMI improves HRQOL and angina symptoms [6].

Hron et al., who examined data from 4,434 middle-aged adults in the Framingham Heart Study, concluded that current smoking was not notably linked with angina (adjusted OR (aOR) = 0.86; 95% CI 0.73-1.01). Conversely, obesity (aOR = 1.77; 95% CI 1.63-3.12), male sex (aOR = 1.45; 95% CI 1.22-1.73), and older age (60-70 years) (aOR = 2.26; 95% CI 1.63-3.12) were major predictors. These results suggest that in this population, age, BMI, and sex have a greater impact on angina risk than smoking [7].

Many studies support a clear correlation between smoking and the start, severity, and course of development of CAD. Early onset of symptoms and multi-vessel disease correlate with higher pack-years of smoking. Those with CAD who smoked were found to be about 10 years younger than those without similar disease severity. Studies of patients with post-myocardial infarction (MI) showed that patients who quit smoking had significantly lower mortality than those who continued smoking (15% vs. 22%; relative risk (RR) = 1.55). Within three years, long-term cessation lowers CAD risk to levels matching those of nonsmokers. These results underline smoking as a modifiable risk factor accelerating CAD and stress the need for cessation for both main and secondary prevention [8]. Mostly through nicotine and tar compounds, smoking cigarettes causes several detrimental effects on the cardiovascular system. Like sympathetic stimulation, nicotine causes tachycardia, higher blood pressure, cardiac output, and myocardial oxygen demand. Smoking simultaneously reduces high-density lipoprotein (HDL) cholesterol, increases platelet adhesiveness, impairs endothelial function, and promotes the penetration of cholesterol-laden lipoproteins into arterial walls. These mechanisms speed up atherosclerosis and lower the red blood cell oxygen delivery. In addition, smoking induces coronary vasoconstriction, stimulates the renin-angiotensin system, and increases inflammatory cytokines (e.g., interleukin-6 (IL-6), tumor necrosis factor-alpha (TNF-α)), contributing to plaque development, instability, and ischemia [8].

A main contributor to cardiovascular disease (CVD), smoking fuels atherosclerosis, thrombosis, and endothelial dysfunction. Free radicals and toxic chemicals in smoke raise oxidative stress, lowering nitric oxide availability and impairing blood vessel dilation. Smoking increases low-density lipoprotein (LDL) and triglycerides while reducing protective HDL, thereby altering lipid profiles. It also causes chronic inflammation with increased markers and adhesion molecules, accelerating the formation of plaque. It also increases platelet activation and alters fibrinolysis, raising the clot risk. Similar damage occurs from both active and passive smoking; even low levels of exposure can elicit these harmful effects, emphasizing that any exposure to smoke increases cardiovascular risk [9].

Using a case-control study, Song et al. investigated whether lipids mediate the relationship between smoking and coronary artery disease (CAD). Of the 2,048 subjects, smoking was clearly linked to higher CAD risk (OR = 1.34). Medication analysis revealed that 17.52% of smoking's impact on CAD was mediated by lipid levels, with HDL-C and triglyceride (TG) contributing 11.16% and 6.36%, respectively. Lipid parameters, specifically TGs, were significantly associated with CAD. Additionally, the TG/HDL-C ratio accounted for 28% of the mediation effect, highlighting lipids as partial mediators in the pathway linking smoking to CAD [10].

In 2,548 patients with suspected stable angina (SA), Schartum-Hansen et al. found that smoking alters the link between plasma choline, troponin T, and AMI risk. High choline was linked in nonsmokers to higher troponin T and increased risk of AMI; in smokers, no such link was found. Particularly low choline (≤8.1 µmol/L) smokers had a six-fold increased risk of AMI. Including choline and its interaction with smoking enhanced risk prediction, thus underlining choline's prognostic relevance by smoking status. Based on individual smoking and metabolic profiles, this could enable customizing of risk assessment and preventive plans [11].

Three months after percutaneous coronary intervention (PCI), 38.3% of patients reported angina. This group included active smokers, who were more likely to experience angina. These patients reported lower baseline Seattle Angina Questionnaire (SAQ) scores (50.2 vs. 69.5) and EQ-5D quality-of-life scores (0.69 vs. 0.84), which remained lower at follow-up (SAQ: 64.0 vs. 95.2; EQ-5D: 0.69 vs. 0.91), indicating that smoking is associated with persistent angina and reduced quality of life following PCI [12].

Lee et al. reported that while passive smoking at home poses a slightly higher risk for angina (RR up to 1.016) than active smoking (RR around 1.007), both smoking and passive smoking increase the risk of angina and CAD. Furthermore, they stated that smoking is responsible for 26.7% of all CVD deaths in Korean adults. The study also revealed that smoking affects CVD differently geographically; urban and industrial areas have a higher risk. These results underline the major part that both active and passive smoking play in CAD [13].

While cocaine use was strongly linked with angina pectoris (aOR 2.21), Patel et al., in a retroactive study of the 2016-2017 National Inpatient Sample (NIS) database including over 58 million hospitalizations, found that nicotine dependence significantly raised the risk of MI (aOR 1.85) [14].

Using National Health and Nutrition Examination Survey (NHANES) data (2013-2018), Chelikam et al. undertook a retroactive cross-sectional study to evaluate the relationship between substance use disorders and CVDs, including angina, MI, and CHD. Among several drugs, smoking cigarettes revealed a particularly strong correlation with CVDs. Compared to those without CVDs, persons with CVDs had noticeably higher rates of smoking (60.47% vs. 40.41%; *P *< 0.0001). In the CVD population, similarly, cigar use (47.05% vs. 35.58%) and smokeless tobacco use (18.53% vs. 14.59%) were more prevalent. Significantly, compared to smoking filtered cigarettes, smoking unfiltered ones was linked with almost twice the frequency of CVD (2.28% vs. 1.10%; *P *< 0.0001). These results highlight the significant role that both traditional and non-traditional forms of tobacco use play in the development of CVDs [15].

Age and gender differences in CAD related to smoking

Our findings indicated a clear age-related pattern in the relationship between current smoking and self-reported angina or CHD. With an OR of 4.50 (95% CI 2.65-7.37), younger smokers aged 18-24 years had the highest odds of CAD, indicating a significant early influence of smoking on heart disease risk. With ORs of 2.05 (25-44 years), 1.93 (45-64 years), and 1.11 (65+), this risk progressively dropped with age but stayed significant across all age groups. These results underline the need for early prevention initiatives since they imply that smoking in younger adults may have a disproportionately greater impact on the development of CAD than in older populations.

Smoking and CAD showed a clear age-related link according to Basnet et al. Although the mean age of CAD sufferers was 56.35 ± 10.5 years, those under 50 were especially susceptible, and smoking was the main risk factor. With a hospital-based study showing an OR of 1.59 (95% CI 1.22-2.07; *P* = 0.001), supporting South Asian studies report 5%-10% CAD prevalence in young adults [16].

Perez et al. found that smoking individuals aged ≤35 years show an OR of 1.59. Smokers with CHD were notably younger than nonsmokers (54.25 ± 9.47 vs. 62.74 ± 9.82 years; *P* = 0.001). This age difference applied to both sexes; female smokers averaged 54.83 years against 63.49 in nonsmokers (*P* = 0.001), and male smokers 54.09 against 62.38 years (*P* = 0.001). In multivariate analysis, age was a major negative predictor of smoking for both men (OR = 0.93, *P* = 0.0009) and women (OR = 0.91, *P* = 0.0009) [17].

Whereas the risk for male smokers was not statistically significant (OR = 0.99; 95% CI 0.94-1.04), our study revealed that female current smokers had a considerably higher risk of CAD (OR = 1.36; 95% CI 1.28-1.44) compared to nonsmokers. This implies that, in line with other studies showing higher vascular vulnerability in women who smoke, smoking might have a more negative impact on women in the development of CAD. These results highlight the need for gender-specific approaches for CAD risk control and smoking cessation.

While men had a higher general CAD incidence, Basnet et al. found that female smokers were more susceptible to its consequences. This is consistent with research on Chinese women who currently smoke, showing increased risk of heart attacks. Although both sexes' current and ex-smoking rates were higher, the stronger effect in women calls for more research, particularly in view of physiological and hormonal variations that might affect vascular damage in women [16]. Smoking was more common among men (77.6%) than women (22.4%; *P* = 0.020) according to Perez et al. Male smokers drank more alcohol (3.08 ± 4.21) than female smokers (1.07 ± 2.49; *P* = 0.001) and were more frequently heavy smokers (39.2% vs. 22.0%; *P* = 0.002). Female smokers (63.3%) experienced depression more frequently than male smokers (49.1%; *P* = 0.018) [17].

Seltzer et al. examined long-term Framingham Heart Study data and found a consistently negative association between cigarette smoking and the incidence of uncomplicated angina pectoris in women over 12-30 years of follow-up. Female smokers had 30%-40% lower rates than nonsmoking women, with risk declining further with increasing cigarette use. Men, on the other hand, showed no appreciable link. Given the prevalence of angina as the most common form of CHD, these results contradict past Framingham studies and the Surgeon General's conclusions, underlining the need to reevaluate presumptions about smoking and angina, especially in women [18].

Recent studies closely match the estimated 420,000 annual first-hand smoking-attributable deaths (SADs) calculated by the Centers for Disease Control and Prevention (CDC), in terms of long-term national trends. Although the prevalence of adult smoking has declined by 40%, the aging population of smokers continues to carry a substantial mortality burden, keeping smoking-attributable deaths (SADs) stable at approximately 450,000 per year through 2030. Subsequently, deaths are expected to decline gradually as the effects of reduced smoking prevalence take hold, highlighting the long-term health consequences of past smoking habits [19].

Genetic, racial, and ethnic disparities in smoking-related CAD

Racial differences in the relationship between smoking and CAD were also observed. While Black non-Hispanic smokers showed a stronger association (OR = 1.21; 95% CI 1.05-1.40), White non-Hispanic smokers had a modestly increased risk (OR = 1.11; 95% CI 1.06-1.16). Hispanic and other racial groups (OR = 1.50; 95% CI 1.35-1.67) had the highest risk observed. These differences could reflect underlying genetic, socioeconomic, or environmental elements affecting smoking-related CAD risk in different racial groups. Targeted treatments addressing these variations could improve cardiovascular outcomes in minority populations.

According to Basnet et al., smoking was clearly associated with CAD in all ethnic groups except the Newar and Dalit. This suggests potential variations in genetics, environment, or lifestyle. Similar to findings in the Han Chinese population, gene-smoking interactions further underscore the need for more ethnicity-specific research [16].

With an eye toward genetic and smoking-related risk, Soliman et al. examined the roles of PIK3C2A and TXNIP gene expression in 215 individuals with acute coronary syndrome (ACS), chronic stable angina (CSA), and controls. PIK3C2A was notably downregulated in CAD, particularly in ACS, compared to CSA, they discovered, while TXNIP was upregulated in STEMI-ACS patients who were non-diabetic smokers with hypertension and hypercholesterolemia. Receiver operating characteristic (ROC) analysis identified PIK3C2A as the most sensitive marker for differentiating ACS from controls, while the combination of PIK3C2A and TXNIP was also optimal for this purpose. Multivariate analysis indicated that PIK3C2A and smoking were independent predictors of ACS, with PIK3C2A, hemoglobin, and cholesterol showing particularly strong correlations with CSA prognosis. These results imply that smoking status, PIK3C2A, and TXNIP together could be biomarkers for angina subtype differentiation and cardiovascular risk assessment [20].

Morita et al. looked at how smoking status plus the ALDH2 genotype affected CAD. Although this combination did not raise the risk of SA, they found that current smoking together with the inactive ALDH22 allele greatly raised the risk of MI and was linked with larger infarct size [21].

Despite similar or higher use of older therapies such as aspirin and beta-blockers, Sonel et al. found that Black patients with high-risk non-ST-segment elevation (NSTE) ACS were less likely than White patients to receive newer, more costly evidence-based treatments, including glycoprotein IIb/IIIa inhibitors, clopidogrel, statins, cardiac catheterization, and revascularization. Although Black patients were less likely to have insurance or primary cardiology care and had higher rates of comorbidities, acute risk-adjusted outcomes were similar between Black and White patients [22].

Among 1,334 patients (630 White, 489 South Asian, 216 Chinese) hospitalized across Canadian centers, King-Shier et al. examined ethnic variations in ACS symptom presentation and treatment. The most common complaint was mid-sternal chest pain, but White patients (33%) more often reported atypical symptoms than South Asians (19%) or Chinese (20%). After adjustment for multiple variables, Chinese patients were less likely to report radiating pain, while South Asians were more likely to report moderate midsternal pain. South Asians with atypical symptoms delayed emergency treatment and were less likely to undergo PCI or CABG. Although no ethnic variations in smoking-related symptom presentation were specifically reported, smoking status was included as an adjustment factor, suggesting its recognized role in ACS risk across all groups [23].

Education and occupational factors

Among those with basic education, the OR for current smoking in individuals with angina or CHD was 1.024 (95% CI 0.9678-1.0832), indicating no statistically significant association. Although the overall percentage of current smokers was lower compared to those with basic education, participants with advanced education and angina or CHD had a statistically significantly higher likelihood of smoking (OR = 1.186, 95% CI 1.1255-1.2449). This could reflect more accurate self-reporting, differences in healthcare access or awareness, or possibly risk-compensation behaviors, whereby individuals with higher education may be more aware of their health conditions yet continue to smoke due to stress or lifestyle factors.

Perez et al. found that smokers were more likely than nonsmokers to be educated (74.6% vs. 55.5%; *P* < 0.001). Among men, 80.3% of smokers were educated compared to 65.3% of nonsmoking men (*P* = 0.008). Although education approached significance in the overall model (OR = 1.69; *P* = 0.0691), it was not significant in analyses specific to women [17].

In a cohort of 3,696 adults, including 221 shift workers, Ryu et al. examined the interaction between smoking and shift work in influencing the risk of ACS. Although education level and age were similar across groups, shift workers were significantly less likely to be female (19.5% vs. 49.8%; *P* < 0.01). The study found that shift work was associated with a significantly higher risk of ACS among nonsmokers (risk ratio 2.26; 95% CI 1.44-3.55; *P* for interaction = 0.01), but not among current smokers (risk ratio 1.05; 95% CI 0.49-2.23). This implies that smoking might either reduce or overwhelm the cardiovascular hazards related to shift work. These results highlight the need for focused cardiovascular prevention programs that give nonsmoking shift workers top priority since they could be more susceptible to the independent consequences of circadian disturbance [24].

Income, smoking behavior, and consequences for public health

Regarding annual income, among those with angina or CHD, there was no appreciable correlation between income level and smoking prevalence. Although the ORs (1.023 and 0.991, respectively) indicated no statistically significant difference, individuals with an income of less than $50,000 had a slightly higher percentage of current smokers (8.4%) than those earning more than $50,000 (4.6%). These results could suggest that although general population smoking behavior is linked with income, it does not significantly vary smoking frequency in the subset of people with CVDs. This could be the result of a ceiling effect whereby other factors (such as nicotine dependence or psychological stressors) play a more dominant role than income alone, or of uniformly high awareness and medical advice regarding smoking cessation across income groups among those with CHD.

Perez et al. also found that smokers more often belonged to higher income groups (27.6% of smokers vs. 16.1% of nonsmokers; *P* = 0.025). Although the income-smoking link was clearer in men (32.7% vs. 22.0%), it did not reach statistical significance (*P* = 0.163), implying a trend toward greater smoking prevalence among wealthier patients with CHD [17].

Kozieł et al. assessed variables linked to ongoing smoking among patients with known CAD. Out of a cohort of 1,034 patients hospitalized for ACS or myocardial revascularization, 28.6% said they smoked in the month before hospitalization, and 54.7% of these said they kept smoking six to 18 months later. Younger people, those exposed to secondhand smoke, and those with a lower socioeconomic level were more likely to be persistent smokers. Successful smoking cessation was linked, on the other hand, to older age, cardiac rehabilitation, cardiologist advice, and better socioeconomic level. These results highlight how important focused post-discharge treatments are to lower smoking rates in patients with CAD [25].

The implementation of smoking bans resulted in a 39% decrease in hospital admissions for cardiovascular conditions (including AMI, angina, and stroke) and a 33% decrease in respiratory admissions during the period the bans affected restaurant settings, according to a Toronto study by Naiman et al. Emphasizing that smoking bans can greatly reduce cardiovascular and respiratory health burdens by lowering tobacco smoke exposure, no similar reductions were observed in control cities or for unrelated health conditions [26].

Taylor et al. underlined how greatly active and passive smoking increase the risk of CVDs, especially CAD and angina. Smoking affects many biological pathways: hematological changes (e.g., increased platelet aggregation), neurohormonal activation (such as raised catecholamines), metabolic abnormalities (like insulin resistance), and endothelial dysfunction via oxidative stress and inflammation. Underlying coronary events, these effects help explain atherosclerosis, plaque instability, and thrombosis. Although some long-term residual risk may persist, smoking cessation can dramatically reduce the incidence of coronary and cerebrovascular disease by up to 50% within two to three years [27].

Using the Rose Angina Questionnaire, Zaman et al.’s PERU MIGRANT study examined angina incidence across rural, urban, and rural-to-urban migrant populations in Peru. Rural residents had a notably higher likelihood of possible or definite angina (aOR = 2.82) than urban dwellers, despite having fewer conventional cardiovascular risk factors, including diabetes and smoking. Particularly, mood disorders, mental health issues were more common in rural areas and were strongly associated with angina symptoms across all groups. These results imply that standardized angina assessment tools may have limited relevance in low-resource rural environments since angina may be influenced more by psychological elements than by classical coronary risk [28].

Limitations

There are several limitations to this study. The BRFSS is subject to reporting errors and recall bias, as it relies on self-reported phone surveys. Excluding individuals with missing data may reduce generalizability and introduce selection bias. Additionally, unmeasured confounding factors could have influenced the outcomes. Those without landlines or unreachable by phone were also excluded. The dataset does not include details on the type, severity, duration, or effects of angina or CAD. As a retrospective observational study, it can only demonstrate associations, not causality.

## Conclusions

This study showed that current smokers had a higher chance of reporting angina or CHD compared to nonsmokers. The association was more noticeable among younger adults, females, and Hispanic and Black individuals. Participants with higher education also showed increased odds, while income did not appear to significantly influence the results. These findings suggest that smoking is an important risk factor for heart-related conditions, regardless of background. Public health strategies should prioritize smoking cessation programs and preventive cardiovascular screening, particularly among high-risk demographic groups identified in this study. Understanding which groups are more affected can help in designing better public health programs. Future research should focus on long-term studies to better understand the relationship between smoking and heart disease and whether other factors like lifestyle or healthcare access also play a role. This can help create targeted strategies to reduce the impact of smoking on cardiovascular health.
